# Hyperbaric oxygen therapy improves colorectal anastomotic healing

**DOI:** 10.1007/s00384-016-2573-y

**Published:** 2016-04-04

**Authors:** G. S. A. Boersema, Z. Wu, L. F. Kroese, S. Vennix, Y. M. Bastiaansen-Jenniskens, J. W. van Neck, K. H. Lam, G. J. Kleinrensink, J. Jeekel, J. F. Lange

**Affiliations:** Department of Surgery, Laboratory of Experimental Surgery, Erasmus MC, University Medical Center, Room Ee-173 Postbus 2040, 3000 CA Rotterdam, The Netherlands; Department of Gastrointestinal Surgery, Peking University Cancer Hospital and Institute, Beijing, China; Department of Surgery, Amsterdam Medical Center, Amsterdam, The Netherlands; Department of Orthopaedics, Erasmus MC, University Medical Center, Rotterdam, The Netherlands; Department of Plastic and Reconstructive Surgery, Erasmus MC, University Medical Center, Rotterdam, The Netherlands; Department of Pathology, Erasmus MC, University Medical Center, Rotterdam, The Netherlands; Department of Neuroscience, Erasmus University Medical Center, Rotterdam, The Netherlands

**Keywords:** Anastomotic healing, Hyperbaric oxygen therapy, Animal model, Perfusion

## Abstract

**Purpose:**

Hyperbaric oxygen treatment (HBOT) has been found to improve the healing of poorly oxygenated tissues. This study aimed to investigate the influence of HBOT on the healing in ischemic colorectal anastomosis.

**Methods:**

Forty Wistar rats were randomly divided into a treatment group that received HBOT for 10 consecutive days (7 days before and 3 days after surgery), or in a control group, which did not receive the therapy. Colectomy with an ischemic anastomosis was performed in all rats. In each group, the rats were followed for 3 or 7 days after surgery to determine the influence of HBOT on anastomotic healing.

**Results:**

Five rats from each group died during follow-up. No anastomotic dehiscence was seen in the HBOT group, compared to 37.5 % and 28.6 % dehiscence in the control group on postoperative day (POD) 3 and 7, respectively. The HBOT group had a significantly higher bursting pressure (130.9 ± 17.0 mmHg) than the control group (88.4 ± 46.7 mmHg; p = 0.03) on POD 3. On POD 3 and POD 7, the adhesion severity was significantly higher in the control groups than in the HBOT groups (*p* < 0.005). Kidney function (creatinine level) of the HBOT group was significantly better than of the control group on POD 7 (*p* = 0.001). Interestingly, a significantly higher number of CD206+ cells (marker for type 2 macrophages) was observed in the HBOT group at the anastomotic area on POD 3.

**Conclusion:**

Hyperbaric oxygen enhanced the healing of ischemic anastomoses in rats and improved the postoperative kidney function.

**Electronic supplementary material:**

The online version of this article (doi:10.1007/s00384-016-2573-y) contains supplementary material, which is available to authorized users.

## Introduction

Colorectal anastomotic leakage (CAL) is the most serious complication following colorectal surgery, causing substantial morbidity and mortality as high as 33 % [1]. With continuous improvements in surgical techniques and perioperative care, the incidence of this complication still varies between 10 and 13 % [2, 3], hardly decreasing in recent decades despite developments in medical science and technology [4].

Regional ischemia has been considered as one of the main causes of anastomotic leakage [5–9]. Poor blood supply and perfusion of the rectal stump and ascending loop of bowel, or prolonged hypoxia are detrimental for wound healing and thus increase the risk of CAL [5, 10, 11]. Poor perfusion delays wound healing processes [12, 13]. The blood supply and perfusion of the preserved bowel, especially at the cutting edge, significantly affects the outcome of patients. Injury to the colon following an ischemic event is due to hypoxia and to reperfusion injury. Bowel ischemia results in hypoxia of the cells. Within 1 h of ischemia, injury in the superficial part of the mucosa is already detectable. Prolonged severe ischemia causes necrosis of the mucosa layer, and lead to transmural infarction within 8 to 16 h [14].

To prevent ischemia, one direct intervention is to provide oxygen. Oxygen is an essential component in tissue repair and wound healing. Oxygen stimulates collagen synthesis, matrix deposition, angiogenesis, epithelialization, and the eradication of bacteria [15–17]. Perioperative use of oxygen has been reported to reduce CAL and improve patient’s outcome after colorectal surgery [18]. Clinical data have shown that perioperative hyperoxygenation reduced the occurrence of surgical site infections [19]. However, consensus in the interpretation of the data in this regard has not yet been reached and the mechanism of the oxygen therapy is still yet to be established.

The use of hyperbaric oxygen therapy (HBOT) is based on the same principle of hyperoxygenation and has been introduced in the treatment of surgical patients as well as in the treatment of patients with chronic wounds [20, 21]. A positive influence of HBOT on anastomotic healing was first reported by Hamzaoglu et al. in 1998 [22]. Although this therapy is widely used in medical practice, its mechanism of action is still poorly understood. Previous study from Attard et al. has suggested that application of HBOT may reduce the production of inducible nitric oxide synthase protein (iNOS) expression [23], which is actively involved during the occurrence of CAL [24]. Many previous studies focused on localized changes such as collagen deposition and MMP (matrix metallopeptidase) activities [25].

The accumulated data supports the hypothesis that HBOT may improve anastomotic wound healing via suppression of pro-inflammatory agents and stimulation of anti-inflammatory agents [26]. This study was carried out to verify this hypothesis in an experimental rat model. HBOT was applied daily to all the rats 7 days prior to a partial colectomy with ischemic anastomosis, until 3 days after surgery. Macroscopic evaluation of anastomotic healing evaluation and immunohistochemistry were performed to investigate involvement of different inflammatory agents.

## Materials and methods

### Animals

Forty male Wistar albino conventional rats, 300–350 g, were purchased from a licensed breeder (Harlan Laboratories, Boxmeer, The Netherlands). All rats were bred under specific pathogen-free conditions and kept under standard laboratory conditions in individually ventilated cages, and had ad libitum excess to water and regular rat chow. The experimental protocol was approved by the local Ethical Committee of Animal Experimentation of Erasmus University Rotterdam.

### Experimental design

The animals were randomly divided into four different groups: control group, 3 days follow-up; control group, 7 days follow-up; HBOT group, 3 days follow up; HBOT group, 7 days follow-up.

The HBOT groups received HBOT for 10 consecutive days from 7 days prior to surgery until 3 days after surgery. Each HBO (hyperbaric oxygen) session consisted of 100 % oxygen under a pressure of 2.4 atm absolute for 90 min in the HBO Test Vessel P1460 [27]. Animals were placed in a large transportation box (ten animals together) during the session. To exclude the possible bias due the HBOT procedure itself rather than the therapy itself, the control groups were also placed in a transportation box for the same time period in the same room, however, without undergoing HBOT.

**Fig. 1:**
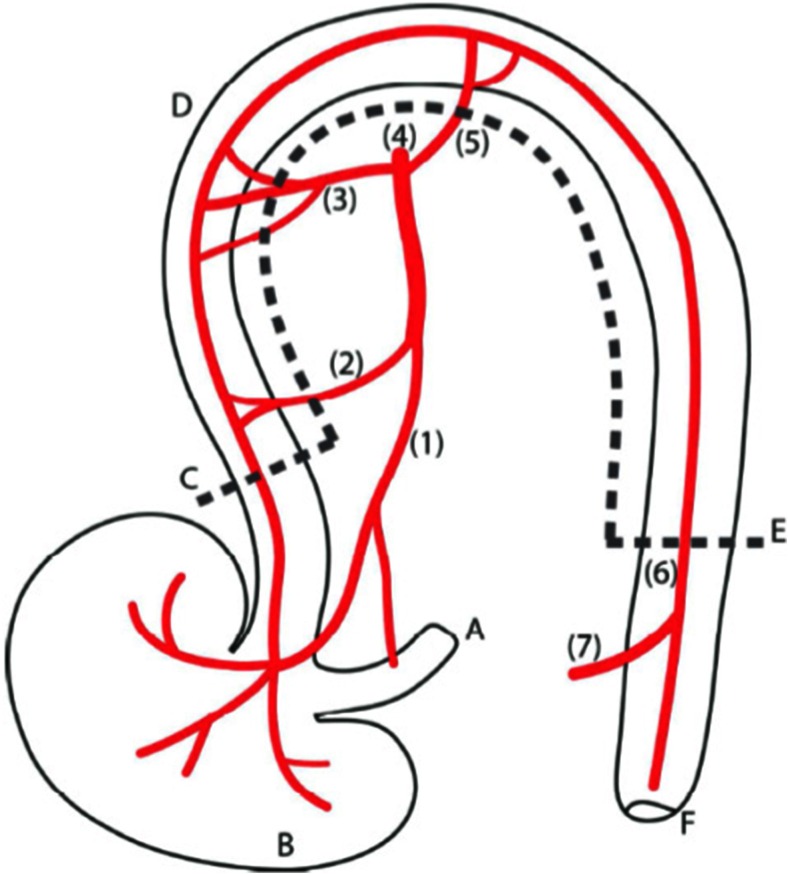
Schematic overview of artery distribution in rat colon, anterior view. (*1*) ileocecocolic artery, (*2*) colic branch of ileocecocolic artery, (*3*) right colic artery, (*4*) cranial mesenteric artery, (*5*) middle colic artery, (*6*) left colic artery, (*7*) caudal mesenteric artery, (*A*) terminal ileum, (*B*) cecum, (*C*) proximal cutting edge, (*D*) colon, (*E*) distal cutting edge, (*F*) anus. Adapted from Wu et al. [28]

On the day of operation, rats were anesthetized using 2 % isoflurane/O2; in addition, preoperatively, 0.05 mg/kg buprenorphine was administered as pain medication. After shaving and disinfecting the abdomen, the abdominal wall was opened through a 5-cm laparotomy. Subsequently, the ileocecocolic arteries, the right colic artery, the middle colic artery, and the left colic artery were ligated (Silkam 4/0, B. Braun, Germany) to create an ischemic anastomosis (Fig. 1). A partial colectomy was performed and proximal and distal ends of the colon were invertedly anastomosed with Dafilon 8/0 (B. Braun, Germany). This model is earlier described, but in short, the colonic segment between 1.0 cm aborally to the cecum and 0.5 cm above the caudal mesenteric artery was resected [28]. The end-to-end anastomosis in all groups was made with 12 continuous sutures. Finally, the abdomen was closed in two layers and the rats were resuscitated with 5 mL of normal saline solution subcutaneously to prevent dehydration. No antibiotics were used. The HBO groups received HBOT immediately after surgery, which continued on postoperative day (POD) 1 and POD 2, and on POD 3 just before the reoperation. During follow-up the rats were weighted and observed daily and had ad libitum access to water and food.

### Assessment of the anastomoses

#### Clinical observation and physical examination

On POD 3 or POD 7, rats were anesthetized again. Anastomotic healing was assessed by observational, physical, and histological examination. The abdomen was checked for signs of anastomotic dehiscence. Abscess formation was scored according to the following scoring system: 0 = no abscess; 0.5 = one very small abscess; 1 = several small abscesses; 2 = medium abscess; 3 = large or several medium abscesses; 4 = one very large or several large abscesses [29, 30]. Adhesion strength and amount were recorded using the Zühlke score [31]. After clinical observation, the anastomotic bursting pressure test (ABP) was recorded in the same way as described previously [28]. In short, the ABP was determined by insufflation of air in the closed segment of the colon, and the first leak of air was noted as the bursting pressure, the location was also noted.

#### Serum measurement

On POD 3 or POD 7, creatinine levels were measured in the serum using the QuantiChrom assay kits (DIUR-500 and DICt-500, Gentaur Europe, Brussels, Belgium).

#### Histopathological evaluation

After the measurement of bursting pressure, a 1-cm-long colonic segment, 0.5 cm on each side of the anastomosic line, was resected and prepared for histopathological examination using standard procedures [28]. HE staining was performed and the anastomotic area of the slides was scored using the Ehrlich and Hunt numerical scale as modified by Phillips et al. [32]. The scoring system evaluated four parameters: inflammatory cell infiltration, fibroblast activity, development of new blood vessels, and collagen deposition. The parameters were graded from 0 to 4 as follows: 0 = no evidence, 1 = occasional evidence, 2 = light scattering, 3 = abundant evidence, 4 = confluent cells or fibers. The samples were scored by three investigators (G.B, Z.W. K.L.), who were blinded for the clinical findings, group allocation, and the treatment. Immunohistochemical staining for iNOS (marker for macrophage type 1; M1, 1:400, Abcam plc, Cambridge, UK, secondary antibody rabbit-anti-rabbit) and CD206 (marker for macrophage type 2; M2, 1:1600, Abcam, secondary antibody rabbit-anti-rabbit) were also performed on anastomotic samples with the same method described previously [24]. After an overnight incubation at 4 °C, the slides were incubated with Envision secondary antibody (DAKO, Glostrup, Denmark). After 30 min, diaminobenzidine (DAKO, Glostrup, Denmark) was used for visualization of antigen-antibody reactivity. Slides were counterstained with hematoxylin.

To determine the positive target cell number on each slide, the same five fields were selected at the anastomotic site on each slide using a microscope with an imaging system (Olympus DP25, Tokyo, Japan), under 20 × 10 magnification (2560 × 1960 pixels). The cell numbers were counted with ImageJ (National Institutes of Health, Bethesda, MD). The average cell number of the selected fields was used for analysis. An M2/M1 index was calculated with the following equitation. The natural logarithm was used to adjust the data to normal distribution.

$$ \mathrm{M}2/\mathrm{M}1\kern0.5em \mathrm{index}=1\mathrm{n}\frac{\mathrm{Number}\kern0.5em \mathrm{of}\kern0.5em \mathrm{C}\mathrm{D}206+\mathrm{cells}}{\mathrm{Number}\kern0.5em \mathrm{of}\kern0.5em \mathrm{iNOS}+\mathrm{cells}} $$

#### Statistical analysis

Statistical analysis was performed with SPSS 21.0 (IBM Inc., Chicago, USA). Data are presented as mean ± standard deviation (S.D.) or as median or as percentage. The Mann-Whitney *U* test, *t* test, and Pearson’s correlation test were used according to proper indications. We used the Levene’s test to test equality of variances. The one-way analysis of variance was performed with the Kruskall-Wallis test for non-parametric parameters. All reported *p* values were two-sided; a *p* value <0.05 was considered to indicate statistical significance.

## Results

### Overall and general observation

In both the control groups and HBOT groups 5 animals died; all deaths are unrelated to anastomotic leakage: 5 colon ischemia, 4 colon ischemic necrosis, and 1 overdosed anesthesia. Postoperative weight loss occurred in all rats without significant difference between the groups.

### HBOT improves clinical parameters

**Fig. 2:**
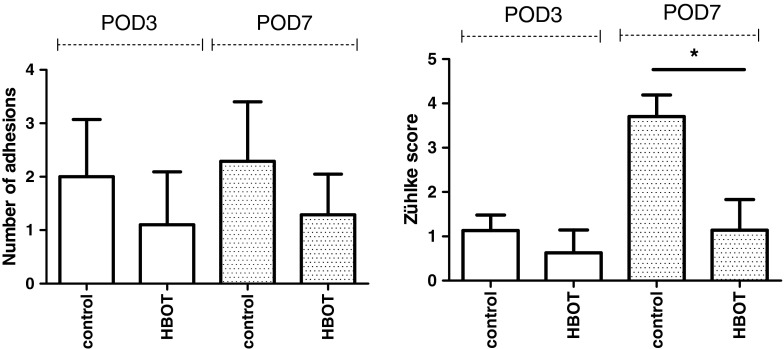
Adhesion score (number of adhesions) and Zühlke score (strength of adhesions) around the anastomosis [31]. The adhesions on POD 7 in the HBOT were significantly less firm; control 3.7 ± 1.1 versus HBOT 1.29 ± 0.8. *indicate significance (*p* = 0.001)

Anastomotic dehiscence was strictly limited to the control groups. The POD 3 group had 37.5 % (3/8) leakage and the POD7 group 28.6 % (2/7) versus a rate of 0 % in both HBOT groups (*p* = 0.021). The number of abscesses between groups was not significantly higher in the control groups (*p* = 0.08) (Table 1). HBOT resulted in significantly less anastomotic adhesions, which were significantly less severe as without oxygen therapy on both POD 3 and POD 7 evaluated with the Zühlke score (Fig. 2).

**Table 1:** Comparison of anastomotic leakage and colon anastomoses abscess rate on POD 3 and POD 7 in the control and HBO groups

	POD 3		POD 7		*p* value
	control	HBO	control	HBO	
Number of rats	10	10	10	10	
Mortality (%)	20 (2/10)	20 (2/10)	30 (3/10)	30 (3/10)	NS
Anastomotic dehiscence (%)	37.5	0	28.6	0	0.02
Colon anastomoses					
Abscess (mean number)	4	2	2	0	0.08

Dehiscence and abscess rate only include surviving animals

**Fig. 3:**
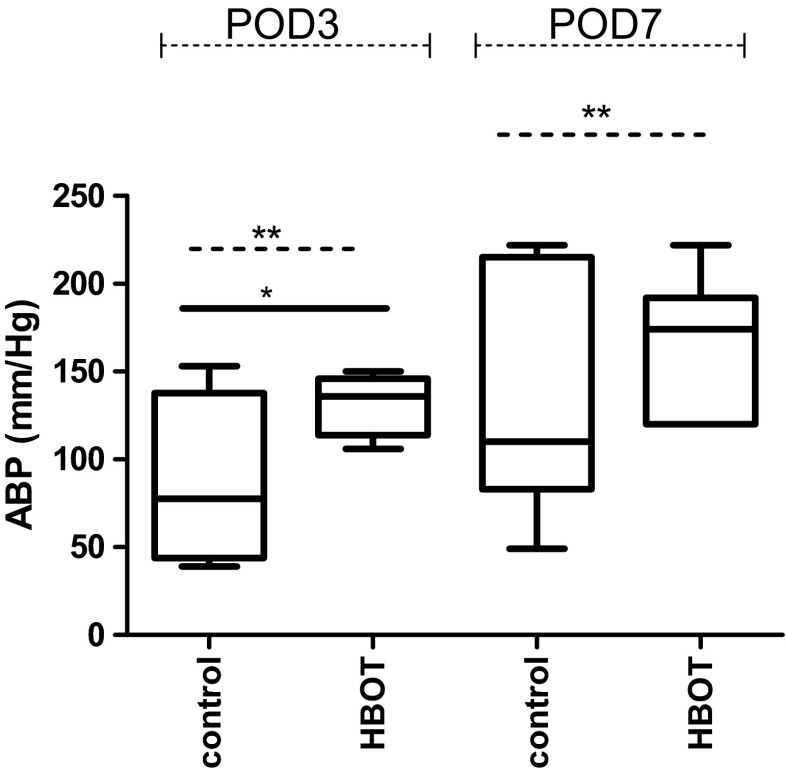
The anastomotic bursting pressure (ABP) in mmHg, was significantly higher on POD3 in the HBOT group 130.9 ± 17.0 mmHg vs. 88.4 ± 46.7 mmHg, p = 0.03. On POD7 we found a trend to an higher ABP in the HBOT group, but not significant (*p =* 0.098), although the variance was significantly lower in the HBOT POD7 group. The variance of ABP between the groups was significantly different and is indicated with the *p* value on the dash line. *indicate p < 0.05, **indicate p < 0.01

On POD 3, the anastomotic bursting pressure (ABP) was significantly higher in the HBOT group than in the control group: 130.9 ± 17.0 mmHg vs. 88.4 ± 46.7 mmHg (*p* = 0.03) (Fig. 3). The variance of ABP in POD 3 in the HBOT group was also significantly lower (*p* = 0.004). ABP was not significantly different between the HBOT group and control group at POD 7 162.4 ± 39.7 mmHg vs. 141.1 ± 73.3 mmHg (*p* = 0.51), but the variance of ABP was significantly lower in the HBO group (p = 0.009).

**Fig. 4:**
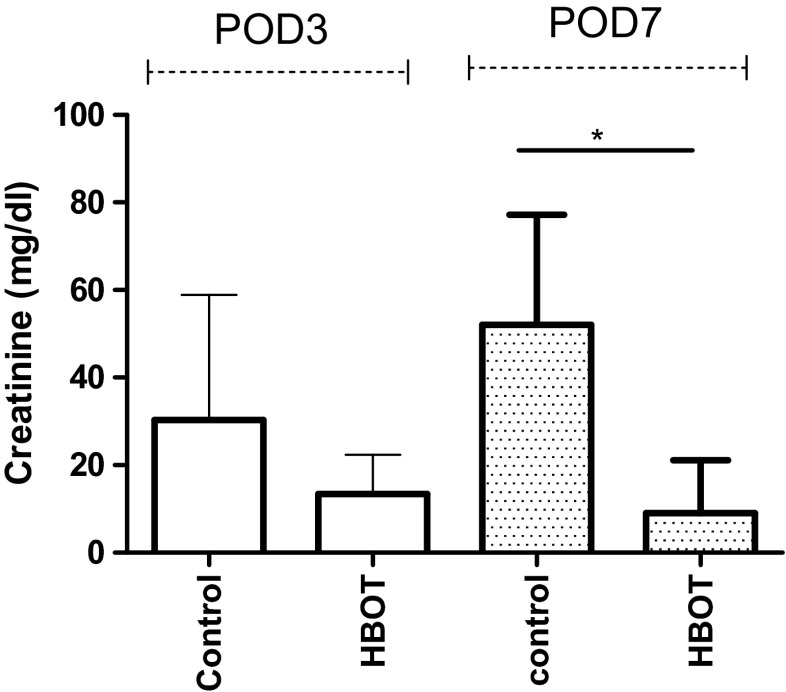
Creatinine levels measured as mg/dl were significantly higher in the control group than in the HBOT group on POD7; 9.0 ± 12.1 vs. 52.0 ± 25.2 mg/dl, *p* = 0.003 (*)

The mean creatinine level in the HBOT group on POD 3 was lower, though not significantly, than in the control group; 13.4 ± 9.0 vs. 30.3 ± 28.6 mg/dl (*p* = 0.07). On POD 7, the creatinine levels were significantly lower in the HBOT group than in the control group, 9.0 ± 12.1 vs. 52.0 ± 25.2 mg/dl (Fig. 4).

### Histology evaluation

**Fig. 5:**
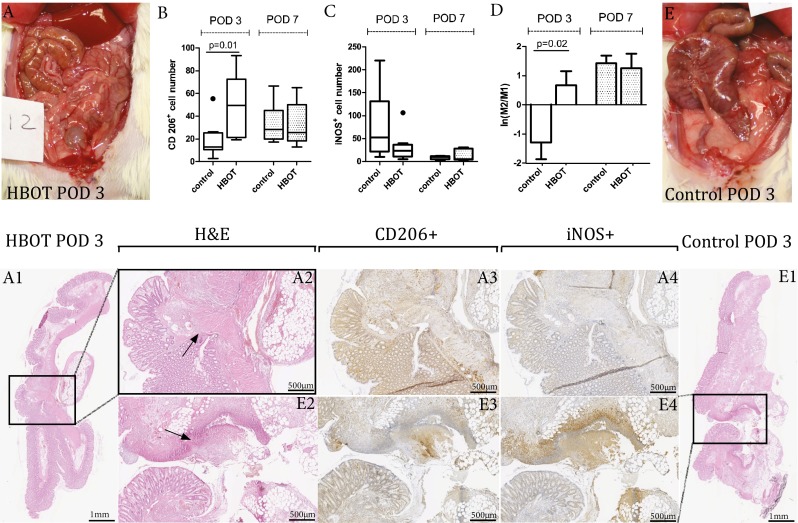
Comparison of macrophage numbers at the anastomotic site on POD 3 and POD 7 of the HBOT groups and control groups. **a** and **e** are representative pictures of an overview of the abdomen at POD 3. **a** A picture of a rat from the HBOT group which has no necrosis or ischemia. *E* shows ischemia of the cecum. **b** Illustrates the CD206+ cell number (indication of M2 macrophages), the *dot above the first bar* indicate an outlier. **c** Illustrates the iNOS + cell number (indication of M1 macrophages), the *dot above the second bar* indicate an outlier. **d** Illustrates the M2/M1 index which is significantly in advantage of the HBOT POD3 group; *p* = 0.02. The *left side* of the image represents an anastomosis of the HBOT POD 3 group, the *left side* of this image is the intraluminal side of the colon. The selected area in *A1* is represented in *A2*. Of the same area the immunohistochemistry staining of CD206+ and iNOS+ is represented in *A3* and *A4*, positive cells are colored with diaminobenzidine. The same is true for the representative histology slides for the control POD3 groups as shown in figure *E1*–*E4*. The *arrows* in *A2* and *E2* indicate the anastomotic line (20 x 10 magnification)

The inflammatory cell infiltration, fibroblast activity, neoangiogenesis, and collagen deposition based on the HE staining were not evidently different between the groups (Supplementary data 1). After 7 days more angiogenesis was observed in the HBOT group. Based on immunochemistry, significantly more CD206+ cells (M2) were present in the HBOT group than in the control group on POD 3; 50.0 ± 28.1 vs. 19.4 ± 16.2 cells, *p* = 0.016 (Fig. 5). Also the M2/M1 index was significantly higher in the HBOT short-term group; *p* = 0.02. The number of iNOS+ cells as a marker for type 1 macrophages (M1, pro-inflammatory marker) was higher in the short-term control group although not significantly different (Fig. 5).

## Discussion

Colorectal anastomotic leakage is a dangerous short-term complication after colorectal surgery and may cause substantial immediate mortality if not treated as soon as possible. In this study, we evaluated the influence of HBOT on ischemic anastomotic wound healing and we found that HBOT improved the wound healing in ischemic colorectal anastomosis as shown with a higher ABP and less firm adhesions. We also observed improved postoperative recovery based on higher creatinine levels in the rats that received HBOT.

When comparing the data from this ischemic colorectal anastomosis model to the standard rat colectomy model [28] and to the other ischemic anastomosis models, the ligation of the arteries of the ascending stump resulted in catastrophic outcomes including evident ischemia at the cecum after surgery and a mortality rate as high as 25 %. This is comparable to the clinical situation in ischemic bowel patients [33]. To our knowledge, this model best mimics clinical outcomes also because most ischemic anastomosis models only cause a lower bursting pressure and localized changes after surgery. Our previous study showed that functional failure of the ascending stump perfusion results in CAL in patients [34]. Impaired tissue perfusion may result from patient-related factors such as smoking inflammatory bowel disease or diabetes [35–37]. Meanwhile, different technique-related factors such as the level of artery ligation and anastomosis configuration may also influence anastomotic perfusion [34]. Our model inflicted a severe ischemic injury to the standard colorectal anastomosis, providing a satisfactory environment for evaluating the influence of HBOT.

Previous studies have reported the beneficial influence of HBOT on anastomotic healing such as increasing ABP [22, 38, 39] and reducing anastomotic adhesions. This was also observed in our study. Moreover, the reduction of intra-abdominal abscess formation indicates an anti-infection effect of HBOT playing an important role in its overall beneficial effect. Because tissue necrosis and ischemia-reperfusion injury caused by the anastomotic ischemia also impair the systematic condition, we expected such beneficial effect of HBOT might also be observed in addition to the localized changes. Though failed to reduce the mortality rate, the HBOT group resulted in better kidney function as a marker for general health on POD 7, indicating a beneficial systematic effect. More importantly, the preconditioning effect remained after cessation of HBOT.

Perioperative oxygen therapy providing 100 % oxygen under higher pressure has been demonstrated to be effective to prevent CAL, but the mechanism is not fully understood. Moreover, it also influences the vascular response. The high concentration of oxygen causes vessel constriction, which has also been reported on HBO therapy cases [40, 41]. Though not fully understood, our preliminary observation found a different response pattern in the HBOT groups after artery ligation (unpublished data), which may eventually influence the blood supply and oxygenation of the tissues after anastomosis construction.

Substantial amount of data demonstrate that the effect of HBOT is not limited to the direct oxygenation [12, 13]. Previous studies showed that HBO therapy increased the ABP even when anastomoses were constructed under contaminated conditions [42, 43]. HBOT has been demonstrated to increase expression of the anti-inflammatory genes as well as influencing the local production of inflammatory cytokines [43], many of which are productions of macrophages. In accordance with that, our data suggest that the influence of HBOT on the inflammatory response via alternatively activating the macrophages. In addition to the increased ABP, the higher M2/M1 index also explains for a reduction of adhesion formation on anastomosis, probably because of earlier onset of regeneration as M2 macrophages enhance the regenerative responses as production of collagen [44, 45].

We investigated the mechanism of the HBOT on colorectal healing using perioperative treatment format which according to the literature has demonstrated the most beneficial effect using perioperative HBOT [39]. We are aware that it is difficult to select the patients who would have a greater risk of ischemic anastomosis and start the treatment preoperatively. However, in some patients, such disposition can be presumed preoperatively. Clinical data remain in query to investigate whether application of HBOT in high-risk patients (e.g., smokers, patients with atherosclerosis, diabetic mellitus, cardiovascular disease, and low colon perfusion during operation due to low blood pressure, blood loss or multiple organ failure) may reduce CAL rate and improve clinical outcome. Whether such application would benefit in a larger scale of patients (i.e. patients without clear risk of CAL) needs further investigation in the future.

## Conclusion

Perioperative hyperbaric oxygen therapy prevents anastomotic leakage in ischemic colorectal anastomosis in the rat. The presence of anti-inflammatory macrophages is associated with the anastomotic healing. Application of HBOT as adjuvant therapy might be useful in the clinic when the patient is critically ill, and given the results of this study, this needs testing in the near future.

## Electronic supplementary material

Below is the link to the electronic supplementary material.

ESM 1 (DOCX 106 kb)
