# Supplementary material for: Hyperbaric oxygen therapy improves colorectal anastomotic healing
Source: Int J Colorectal Dis. 2016 Apr 4;31:1031–8. doi: 10.1007/s00384-016-2573-y (PMC4834105; doi:10.1007/s00384-016-2573-y)

Supplementary data:

Comparison of inflammation, fibroblast formation, angiogenesis and collagen deposition at the anastomotic site in the control and HBOT groups. There were no significant differences between the groups at any time point.


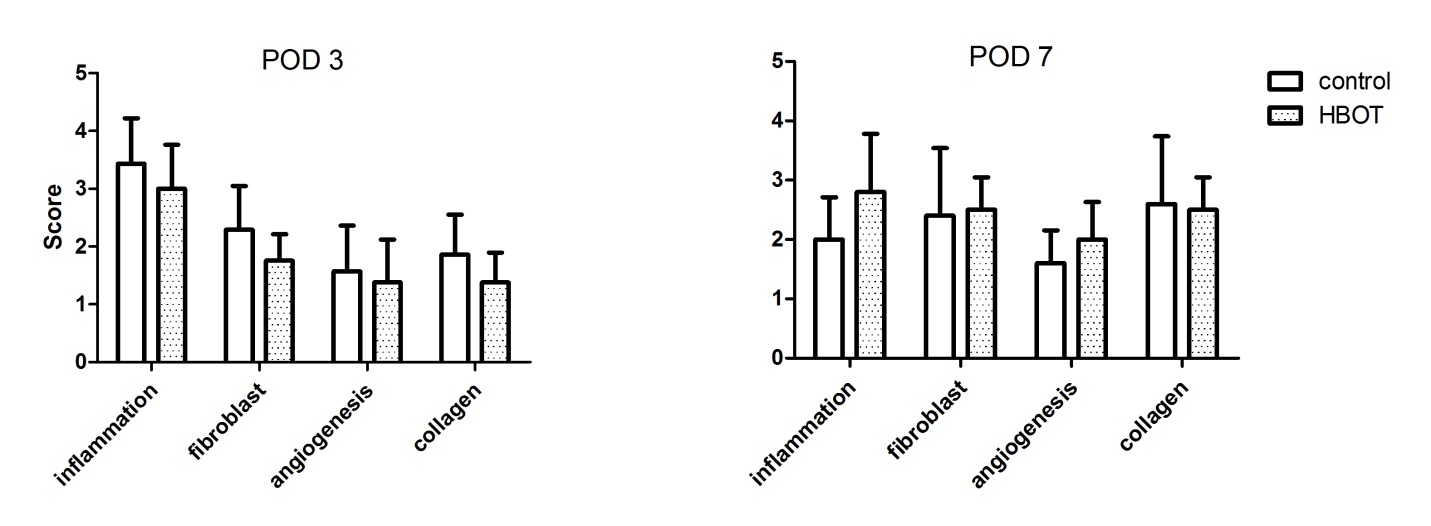

Supplement: Supplementary file 1 — (DOCX 106 kb) [file 384_2016_2573_MOESM1_ESM.docx]
